# The Prevention of Fire

**Published:** 1913-01-25

**Authors:** W. J. Dobbie

**Affiliations:** Physician-in-Chief, Toronto Free Hospital, Weston.


### THE PREVENTION OF FIRE.

By Dr. W. J. DOBBIE, Physician-in-Chief, Toronto Free Hospital, Weston.

[Read before the Canadian Hospital Association.]

Having achieved the rather unenviable notoriety of having had the hospital with which I am connected burned to the ground, your Executive Committee generously considered that I should be given as well the desirable distinction of an opportunity to address you on this occasion on the subject of "The Prevention of Fire." The subject is perhaps not only timely but of more or less general interest to those who may have the responsibilty of safeguarding the lives of the sick and the helpless in institutions of various kinds.

I have therefore the more willingly, on account of the importance of the subject, undertaken to compile some information, on the one hand as to things which ought not to be done, and some on the other concerning things that ought to be done if the institutions in which we live and work are to be reasonably safe from the devastating ravages of the most destructive agent known as "fire."

For the former of these—namely, things that ought not to be done, it may be presumed that I am myself a sufficient authority, while for the latter—things that ought to be done—I may say that I have freely sought the assistance of others, among whom were many of yourselves. And I trust that the combined result may be found of assistance to every hospital superintendent in the land.

### The Story of a Serious Fire.

At the outset then perhaps it may be pardonable for me to relate to you briefly our own experience, when the Toronto Free Hospital was destroyed by fire, since it illustrates very forcibly some of the points to be subsequently made. In the hospital at the time were some 110 patients, of whom about fifty were confined to bed and practically helpless. These latter, further, as fortune would have it, were located in the section destroyed, about twelve being on the ground floor and the remaining thirty-six on the first floor.

At about 2.15 a.m. the attention of the senior night nurse was drawn by one of the patients to the fact that he smelled smoke. She immediately ran to the diet kitchen, sitting-room, main kitchen, etc., to see if by any chance the smoke arose from some trifling cause. Finding everything as usual she ran to inspect the furnace-room in the basement, and discovered even before she reached it that the trouble was centered in that locality. Here the nurse, without time for second thought, displayed most excellent judgment in that (1) she realised at once that the cause of the smoke was too serious for her to attempt to handle alone, and (2) in immediately, without alarming anyone else, running to awaken the nurses and employees in their respective cottages. She thus had at hand in a few moments some thirty helpers, to each of whom had been assigned months previously his or her particular duty in just such an emergency. And I may perhaps pause here to remark that one of my greatest regrets is that I was not privileged to witness what I am told was a splendid sight to see—namely, employees and nurses rushing out into the night scantily clad, two in this direction, three in that, and so forth, each to reach his post of duty with the least possible delay.

The rule had always been that patients were to be removed first, and as each man had his particular wards assigned to him it was but a very few minutes before every patient had been safely removed. There was no confusion and little excitement. No one was injured in any way, and no mistakes were made, though the corridors were dense with smoke, and the extent of the fire at this time could only be judged by the reflection from the flames as they rapidly made their way from the basement to the ground floor. Nothing of any value was saved except some mattresses and bedding which the nurses threw out of the windows to be used immediately for the rescued patients.

### Improvising Breakfast.

Two interesting events may also be mentioned: (1) The roll-call at 3 a.m., at which every patient, nurse, and employee was accounted for; (2) at 7 a.m also the busy scene of providing breakfast, with improvised equipment from impoverished stores, served but to emphasise the surplus of good fellowship which nothing but common disaster or adversity seems able to discover. And if one were able without seeming to boast to refer to the faithfulness and unselfishness of one's employees, it would only be expressing a natural pride which one takes in finding qualities in men and women hitherto unseen or even imagined.

It is very gratifying indeed, ladies and gentlemen, to know that these things were all done by ordinary employees and nurses, just such as you all have in your respective institutions, and done also without previous experience, as the result simply of preliminary instruction. It tends, as I take it, to strengthen the confidence of the public in institutions and to relieve from the superintendents much of their load of care, inasmuch as what men and women have done men and women could do, and will do doubtless when the occasion again arises, whether it be in your institution or in mine.

To come, however, to the subject of the paper, I may say that a list of questions was sent to some hundred and fifty hospitals in Canada and the United States. The questions are as follows: (1) How many patients' beds are there in your hospital? (2) Do you employ a night watchman? (3) If so, has he any other duties besides those of night watchman? (4) What fire appliances are on hand in your hospital? (5) What is the nature of your water-supply for fire purposes? (6) Do you have "fire drill"? (7) If so, of what does it consist? (8) What measures have you adopted to secure the safe removal of patients in case of fire? (9) Have you ever had a fire? If so, what have you learned from it?

(10) What are your special ideas on the subject of fire?

A large number of replies were received, and from these the material in the subsequent portion of this paper has been compiled.

### Hospital Construction.

With a degree of unanimity hardly to be expected a large majority have pronounced in favour of fireproof hospital buildings. No doubt this has been due in a large measure to the fact that the writers have from experience been brought to realise the insecurity of buildings otherwise constructed.

One writer says: "All buildings for the reception of sick, injured, or insane people should be so constructed as to be absolutely fireproof.

"In all hospitals of more than one flat there should be an elevator so placed and so constructed that a fire in the neighbourhood of or in the elevator well would be an absolute impossibility.

"Pavilions should be so arranged that they could be at once shut off from each other by fire-doors. In the construction of a fireproof building all partitions should be of brick, terra-cotta, stone, or metal. The floors should be cement carried on steel beams. Covering the cement there should be a layer of some of those fireproof compositions. A wooden floor should not be laid in the wards of any hospital. The plaster on the walls and ceilings should be spread upon expanded metal lath. The only woodwork, therefore, that should be found about a hospital would be that necessary to make the plainest form of door, and these in turn should be covered with some metal sheeting. A hospital so constructed has nothing whatever that could burn but the bed-clothing that may be over the patient. I may go the length of saying that the provincial Governments for all the provinces should regulate by legislation the construction of hospitals so as to make them conform with the foregoing idea."

This I think fully covers the matter of hospital buildings. In these days when factories, offices, and warehouses are being so constructed, it is little less than criminal to house the sick, the helpless, or the insane in buildings of an inflammable nature. The fact remains, nevertheless, as may be judged from the replies received, that the fireproof hospital building is just as rare as is the Board of Trustees alive to the need of such a structure.

### Good and Bad Fire Appliances.

On the subject of fire appliances one is at once impressed by the regularity with which certain equipment has been reported as being in use. The most common are: (1) Hydrants and hose; (2) Fire pails; (3) Chemical fire-extinguishers.

This is of interest inasmuch as it illustrates the extent of the average idea as to what constitutes fire protection. These articles comprise the ordinary equipment which one sees in almost any building, and while no doubt at times they have been found extremely useful, as I can attest from personal experience, yet I think it may also be safely asserted that they but too frequently serve merely to lull the occupants into a feeling of false security. In our own fire to which I have referred all of these things were provided, but were of absolutely no use inasmuch as the site of the fire could not be reached. What we most needed was an axe and a saw, and as fortune or bad management would have it, all of these were neatly stored in the basement where the fire originated. These useful articles should be located at various points throughout the buildings, and should be plainly labelled "For fire only."

Among other appliances mentioned were: Ropes, ladders, stretchers, blankets, etc. In connection with the latter a most ingenious and valuable suggestion was that of a woollen blanket and a bath-tub filled with water, at nightfall. This equipment on each flat might prove most useful in many ways in quenching an incipient blaze.

Fire escapes are nearly always mentioned as being of great value. My own opinion is that as ordinarily constructed, located, and used they are greatly overrated. To be of much service a fire escape should be liberal in its dimensions, so that even those unskilled in gymnastics might have some chance of descending by them. They should, moreover, be located so as to be in constant use. Our fire escapes were not used at all at the time of the fire, though conveniently located, and the reason given by employees and others was that in carrying out a patient they did not care to risk the route with which they were not familiar. Fire escape exits, if in daily use, could be made of great value, but otherwise they will only be used when absolutely necessary, and then accidents are likely to occur. It may further be noted that when fire escapes are placed on the outside of a building they should be placed against a blank wall, and should not, as is often the case, pass in front of windows.

A much better arrangement, however, is to provide fireproof stairways, so arranged with automatic fire-doors that the fire may be prevented from spreading from one floor to another.

It would also seem to be desirable that some reference should be made to the value of the automatic sprinkler system, which could at least be installed in such portions of every hospital building as are most likely to be the site or origin of a fire, such as furnace rooms, main kitchens, diet kitchens, laundries, etc. The construction of such a system is such that when a temperature of say 155° F. is produced a connecting link is fused and a shower of water released at the point at which the fire is located.

In connection with these systems there can also be installed an automatic alarm, which may be either in the form of a special gong or connected to the ordinary annunciator. These systems may be connected with city waterworks supply, gravity tank erected on a tower above the highest point of the building, or with a fire pump.

*(To be continued.)*

[It is remarkable that the chief point emphasised by Dr. Dobbie is that insisted on by The Hospital when the Leeds fire broke out: the importance of every institution at which a fire occurs publishing the lessons which have been learnt from it.—Ed.]

